# Exposure to particulate matter modifies the pancreas extracellular/intracellular HSP70 ratio in high-fat diet treated mice: a marker of the diabetes development risk

**DOI:** 10.1186/1758-5996-7-S1-A147

**Published:** 2015-11-11

**Authors:** Pauline Brendler Goettems Fiorin, Bethânia Salamoni Grochanke, Fernanda Giesel Baldissera, Analu Bender dos Santos, Paulo Ivo Homem de Bittencourt, Mirna Stela Ludwig, Claudia Ramos Rhoden, Thiago Gomes Heck

**Affiliations:** 1 Unijuí, Ijuí, Brazil

## Background

Evidences highlights exposure to fine particulate matter (PM2.5) as a risk factor for development of type 2 diabetes (T2D), especially in high-fat diet (HFD) fed mice. Under stressful conditions, cells respond by synthesizing a suite of intracellular stress response proteins, that plays a fundamental protective role in metabolic disorders, the 70 kDa family of heat shock proteins (iHSP70). However, while iHSP70 presents anti-inflammatory proprieties, their extracellular levels (eHSP72), presents pro-inflammatory roles. We propose to investigate the effects of HFD+PM2.5 association in the HSP70 status, and this relation to the risk of developing T2D.

## Materials and methods

Male mice (n=20) were fed standard chow or HFD for 12 weeks, and then, were randomly exposed to daily nasotropic instillation of low doses of PM2.5 (5 μg/10 μL) or saline solution for additional 12 weeks, performing four groups (n=5): Control, PM2.5, HFD and HFD+PM2.5. At the end of the study, we assessed the eHSP72 plasma concentration by highly sensitive EIA method and the pancreas iHSP70 expression by immunoblot analyses. Assuming the ratio R=[eHSP72]/[[iHSP70]=1 for the Control group, the R was analyzed separately for each group. R values between 0.0 and 1.0 indicate a predominantly anti-inflammatory (cytoprotective) status, conversely R values higher than 1.0 denotes pro-inflammatory response. Statistical analysis was developed using ANOVA and post hoc Tukey's test.

## Results

**Figure 1:**
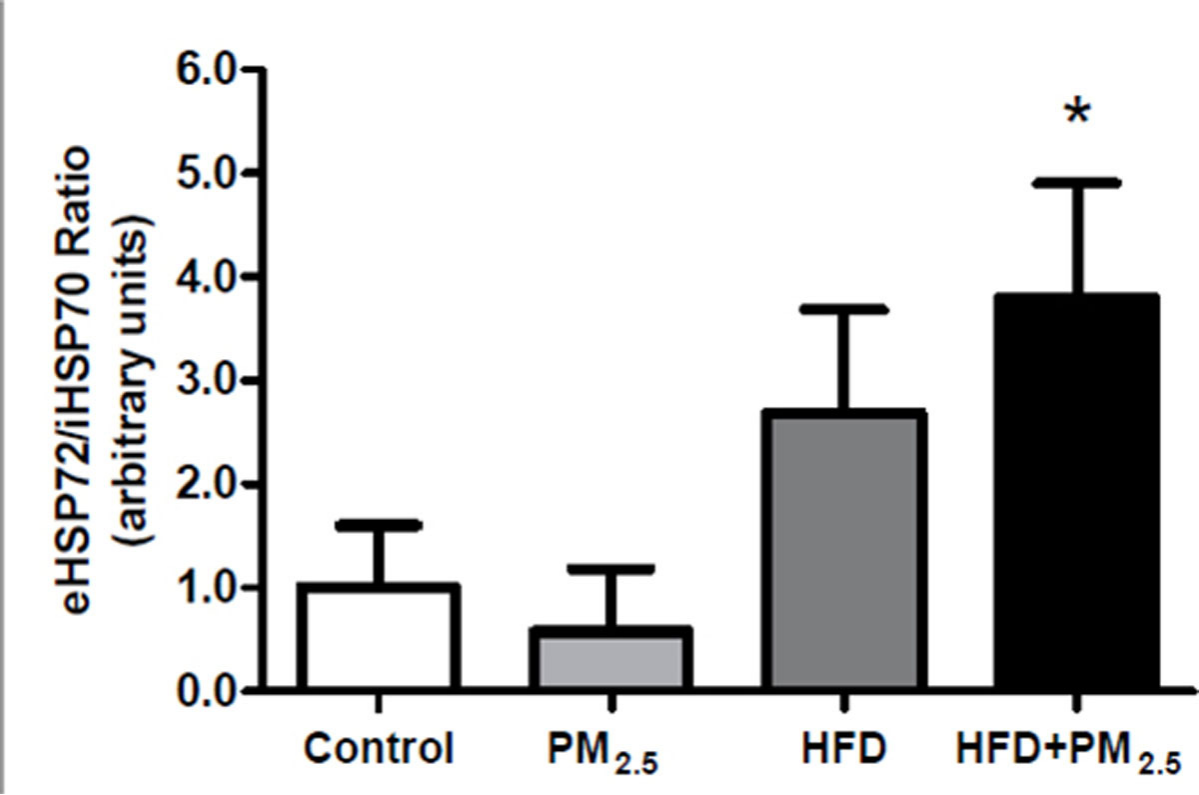
Effect on [eHSP72]/[iHSP70] ratio, mean ± SD. *Different from Control and PM_2.5_ group. P=0.005.

There were no alterations on eHSP70 plasma concentration (P=0.2798). Pancreas iHSP70 expression was lower in HFD groups (Control 1.0±0.4; PM2.5 1.2±0.6; HFD 0.2±0.1; HFD+PM2.5 0.1±0.1 arbitrary units of HSP70; P=0.0007), already indicating low cytoprotection in these groups. Furthermore, the [eHSP72]/[iHSP70] ratio was increased 3.8 fold in HFD+PM2.5, as shown in Figure 1, denotes pro-inflammatory condition.

## Conclusion

Our study showed that HFD consumption decreases the pancreatic cells defense, and this condition associated with sub-chronic exposure to PM2.5 promotes imbalance in HSP70 status, described by the [eHSP72]/[iHSP70] ratio, indicating an immunoinflammatory status, which can be determinant to trigger a chronic pro-inflammatory state that leads to insulin resistance. These data provides evidence for an important interplay between environmental and dietary challenges that may potentiate the development of T2D, highlighting HSP70 status as a biomarker of this condition.

